# Clinical efficacy of exercise in the treatment of post-COVID-19 syndrome: a systematic review and network meta-analysis

**DOI:** 10.3389/fphys.2025.1656713

**Published:** 2025-12-18

**Authors:** Shaojie Du, Zeyu Cui, Xiangqian Xu, Te Liu, Jie Ye

**Affiliations:** 1 Shanghai University of Traditional Chinese Medicine, Shanghai, China; 2 Department of Orthopedics and Traumatology, Longhua Hospital Shanghai University of Traditional Chinese Medicine, Shanghai, China; 3 Shanghai Geriatric Institute of Chinese Medicine, Shanghai University of Traditional Chinese Medicine, Shanghai, China; 4 Department of Respiratory Medicine, Longhua Hospital Shanghai University of Traditional Chinese Medicine, Shanghai, China

**Keywords:** post-COVID syndrome, exercise therapy, clinical trials, netword meta-analysis, PCS

## Abstract

**Background:**

Post-COVID-19 syndrome (PCS) describes a constellation of persistent or new symptoms lasting beyond the acute phase of SARS-CoV-2 infection. Emerging evidence suggests that exercise is a cost-effective and accessible intervention that may enhance pulmonary function, improve cardiopulmonary circulation, regulate emotional status, and alleviate symptoms of PCS. However, robust evidence supporting the efficacy of exercise therapy in PCS remains limited. This systematic review and meta-analysis aimed to elucidate the therapeutic potential of exercise therapy in PCS.

**Method:**

A search of the PubMed, Embase, Web of Science, and Ovid databases up to March 25, 2025 yielded 33 randomized controlled trials (with 2,895 participants) for meta-analysis.

**Result:**

The results showed that exercise therapy significantly improved the multi-dimensional outcomes of patients with PCS. Bayesian network meta-analysis indicated that the combination of aerobic exercise and respiratory muscle training had the best effect on lung function. Multimodal exercise significantly improved the results of the six-minute walk test, the dyspnea score, and peak oxygen uptake. Mental Health and Mental Component Summary scores improved significantly in the group that received exercise therapy (P<0.01).

**Conclusion:**

The results of this meta-analysis confirm that exercise can significantly improve quality of life and the emotional state of patients with PCS. They also provide evidence for a treatment strategy in patients with post-COVID-19 sequelae.

**Systematic Review registration:**

https://www.crd.york.ac.uk/PROSPERO/#myprospero, identifier CRD420251034187.

## Introduction

Post-COVID-19 syndrome (PCS) is a multisystem disorder in which a subset of patients develop persistent or new symptoms following the acute phase of severe acute respiratory syndrome coronavirus 2 (SARS-CoV-2) infection. These symptoms manifest as organ dysfunction that cannot be explained by alternative diagnoses and often involve protracted fatigue, cardiopulmonary compromise, and neurocognitive impairment (Pierce et al., 2022; Yong, 2021; Ceban et al., 2022). SARS-CoV-2 is classified as a coronavirus because its surface protrudes like a crown. In most cases, its symptoms are similar to those of the common cold, namely, coughing and fever. Severely ill patients present with typical respiratory symptoms, including severe breathing difficulties, hypoxemia, and acute respiratory distress syndrome, which may progress to a systemic inflammatory storm and even to sepsis or shock (Yuce et al., 2021). In the post-acute phase following resolution of symptoms or during a prolonged period of immunocompromise, individuals may experience a persistent or novel constellation of symptoms, including fatigue and malaise, palpitations and chest pain, anorexia and diarrhea, anxiety and depression, cognitive decline and memory impairment, and concurrent symptom clusters. The incidence of PCS ranges from 10% to 35%, being more common among the older adult with chronic underlying diseases and in individuals with weakened immune systems. There is some evidence to suggest that PCS is more common in women (Yong, 2021; Majumder and Minko, 2021).

In the absence of any specific diagnostic criteria, PCS is usually diagnosed based on the medical history and after exclusion of other conditions. There is no cure for this syndrome, and most of the treatments are symptomatic, such as drug therapy to relieve airway obstruction, alleviate pain, and combat anxiety. Behavioral cognitive therapy improves “brain fog,” cognitive impairment, and memory loss. Other non-pharmacological therapies may also be used for symptomatic relief (Habas et al., 2020; Kevadiya et al., 2021; Bamji, 2021). Exercise therapy, which includes aerobic exercise (AE), resistance training (RT), and respiratory muscle training (RMT), is a conservative, simple, and cost-effective treatment with several advantages, including a choice between a wide range of exercises and methods and exercises of varying intensity. Exercise is an important component of rehabilitation medicine, and our previous research has confirmed that it can significantly improve symptoms in patients with lumbar disc herniation and enhance their quality of life (Du et al., 2025). In a clinical cohort study, Tucker et al. found that exercise therapy reduced the incidence of cardiovascular disease, improved cardiopulmonary function, and enhanced the self-protection ability of the heart (Tucker et al., 2022). Smart et al. demonstrated that resistance exercise can enhance cardiopulmonary function, muscle mass, and functional capacity in healthy older adult individuals aged over 60 years. Furthermore, AE has been shown to significantly improve cognitive flexibility, working memory, and inhibitory control in healthy middle-aged and older adult (Ye et al., 2024). The combination of multiple exercise therapies may make the clinical effect more significant (Smart et al., 2022). Exercise therapy has not only brought about improvements in both organic and functional diseases, but also made contributions to the management of psychiatric disorders (Abdullahi et al., 2024). A cohort study by Harvey et al. found that regular exercise protected against symptoms of depression (Harvey et al., 2018), while Herring and Meyer found that RT and RMT could alleviate anxiety (Herring and Meyer, 2024).

There is still no systematic summary of its validity and accuracy in PCS. And traditional pairwise meta-analyses could not adequately compare different exercise modalities simultaneously, thus we employed a Bayesian network meta-analysis to integrate direct and indirect evidence across interventions. Therefore, this meta-analysis was performed to evaluate the clinical efficacy of exercise therapy in patients with PCS and its auxiliary role as a non-pharmacological treatment. Our analysis of three exercise methods (AE, RT and RMT) confirmed their feasibility and safety in multiple rehabilitation treatment models.

## Materials and methods

### Search strategy

The PubMed, Embase, Web of Science, and Ovid databases were searched through to 25 March 2025 for relevant clinical studies. This research is registered on PROSPERO (CRD420251034187).

### Eligibility criteria

**FIGURE 1:**
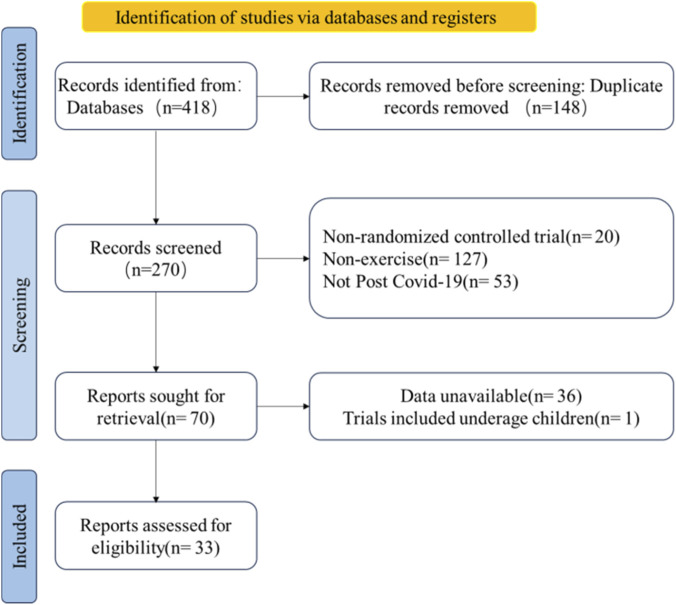
Flowchart showing the study selection process.

Studies were eligible for inclusion in the meta-analysis if they were randomized controlled clinical trials, the disease under investigation was PCS, the main intervention was exercise therapy, outcomes were described, and the publication language was English. No changes to the drug regimen during the month before the intervention or during the intervention were permitted. Furthermore, no restrictions were set for comparison countries. Eligible studies were required to include specific outcome indicators, such as lung function, peak oxygen uptake (VO_2_), and the modified Medical Research Council (mMRC) score, for evaluation of the effectiveness of exercise therapy in PCS. Figure 1 shows the process used to select studies for inclusion in the meta-analysis. Any differences were resolved by discussion until consensus was reached. No grey literature search was conducted because our focus was limited to peer-reviewed randomized controlled trials to ensure data quality and consistency. The detailed search strategy is shown in Table 1.

**TABLE 1:** Search strategy used to identify relevant studies in four major databases.

Database	Number	Search strategy
PubMed	-	(((Randomized Controlled Trial [Publication Type]) OR (Clinical Trial [Publication Type]) OR (Observational Study [Publication Type])) AND ((Exercise [Title/Abstract]) OR (Exercise [MeSH Major Topic]) OR (Physical Therapy [Title/Abstract]) OR (exercis*[tiab]) OR (physical activit*[tiab]) OR (training [Title/Abstract]))) AND ((Post-COVID Syndrome [Title/Abstract]) OR (Post-COVID-19 [Title/Abstract]) OR (Post-Acute COVID-19 Syndrome [Title/Abstract]) OR (Post COVID-19 Condition [Title/Abstract]) OR (Chronic COVID Syndrome [Title/Abstract]) OR (long haul* COVID [Title/Abstract]) OR (post-acute sequelae of SARS-CoV-2 [Title/Abstract]))
WOS	#1	TS=(“Long COVID” OR “Post-COVID Syndrome” OR “Post-Acute COVID-19” OR “PASC” OR “Chronic COVID”)
	#2	TS=(“exercise therapy” OR “physical activity” OR “training”)
	#3	DT=(“Clinical Trial” OR “Proceeding Paper”)
	#4	#3AND#2AND#1
Ovid	1	exp Post-COVID Syndrome/or (Long COVID or Post-Acute COVID-19 Syndrome or PASC).mp
	2	exp Exercise Therapy/or exp Exercise/or exp Rehabilitation/or (exercis* or “physical therap*” or rehab*).mp
	3	exp randomized controlled trial/or exp clinical trial/
	4	1and2and3
Embase	#1	“post covid-19” OR “long covid”/exp OR “post acute covid 19 syndrome”/exp OR “long haul* covid”:ti,ab OR “pasc”:ti,ab
	#2	“exercise”/exp OR “exercise therapy”/exp OR “rehabilitation”/exp OR “physical activit*”:ti,ab OR “aerobic training”:ti,ab OR “resistance training”:ti,ab
	#3	“randomized controlled trial”/de OR “clinical”/de
	#4	#1AND#2AND#3

### Risk of bias assessment

Two researchers independently evaluated the methodological quality of the included randomized controlled trials based on the Cochrane risk of bias criteria and classified each quality item as low risk, high risk, or uncertain risk. The seven items of bias in each trial evaluation included randomized sequence generation, allocation concealment, subject and personnel blinding, outcome assessment blinding, incomplete outcome data, selective reporting, and other biases.

### Extraction of data

The reviewers extracted the following information from the included studies: authors, publication year, trial design, type of intervention, number of study participants, age and sex distribution, and study outcome indicators. If there were multiple subgroup outcomes under the same outcome measure in the study, we handled them using the sub-combination and union formula. For subgroups that performed multiple exercises (e.g., AE and core training), the inverse variance weighting method was used for merging. Subgroup data were merged by combining subgroup A (sample size N_1_, mean M_1_, standard deviation SD_1_) and subgroup B (N_2_, M_2_, SD_2_). If there were multiple subgroups of data that needed to be merged, the following formula was used:

$$N=N_{1}+N_{2}$$

$$M=\frac{N_{1}M_{1}+N_{2}M_{2}}{N\;}$$

$$SD=\sqrt{\frac{\left(N_{1}-1\right){)SD}_{1}^{2}+\left(N_{2}-1\right){SD}_{2}^{2}+\frac{N_{1}N_{2}}{N}(M_{1}-{\left.M_{2}\right)}^{2}}{N-1}}$$

First, the data for two of the subgroups were merged, after which the data obtained were merged with data for the third subgroup. Any differences in opinion among the reviewers were resolved by consensus or by consultation with a third-party reviewer.

### Data analysis and statistical methodology

The data were analyzed using Review Manager 5.4. Continuous data are summarized as the mean difference, which was the 95% confidence interval (CI). Heterogeneity was evaluated using the I^2^ test. In accordance with the recommendation in version 5.1.0 of the Cochrane Handbook, we defined I^2^ as follows: <25%, mild heterogeneity; 25%–50%, moderate heterogeneity; and >50%, severe heterogeneity. We used a random effects model when heterogeneity was >50% and a fixed effects model when it was ≤50%. For studies with high heterogeneity, we used Stata14 software for the sensitivity analysis to evaluate the stability of the results. When the number of studies of the same indicator was sufficient, we performed a network meta-analysis using the Bayesian method and the GeMTC package in R 4.1.0 (R Foundation for Statistical Computing, Vienna, Austria). This analytical framework is helpful for comparing the results obtained by various research groups and enhancing our understanding of the effectiveness of various intervention measures.

This study protocol was prospectively registered on PROSPERO (CRD420251034187).

## Results

### Literature screening

As shown in Figure 1, the database generated 418 unique records, 148 of which were excluded for being duplicates. After application of the screening criteria, a further 200 studies were excluded, leaving 70 potentially relevant studies. Thirty-six of these studies were found to have missing data and were excluded, as was one study in young children, leaving 33 studies for inclusion in the meta-analysis (Del Corral et al., 2025; Romanet et al., 2022; Rodriguez-Blanco et al., 2023; de Moraes Sirydakis et al., 2024; Sánchez Milá et al., 2024; Rayapuraju et al., 2025; Paneroni et al., 2024; Lai et al., 2024; Okan et al., 2022; Elyazed et al., 2024; Jorge et al., 2025; Kerling et al., 2024; McNarry et al., 2022; Sánchez-Romero et al., 2025; Bileviciute-Ljungar et al., 2024; McGregor et al., 2024; Gaudreau-Majeau et al., 2025; Sick et al., 2025; Abo Elyazed et al., 2024; Bai et al., 2024; Özlü et al., 2022; Kogel et al., 2023; Dwiputra et al., 2024; Gomes Dos Santos et al., 2024; Çelik et al., 2024; Espinoza-Bravo et al., 2023; Cunha et al., 2024; Besnier et al., 2025; del Corral et al., 2023; Fares et al., 2023; Jimeno-Almazan et al., 2022; Jimeno-Almazan et al., 2023; Barz et al., 2024).

### Characteristics of the included studies

Supplementary Table S1 shows the characteristics of the 33 included studies (involving 2,895 participants). All were published between 2022 and 2025. Two reviewers summarized the intervention methods used in the studies as follows: none (no exercise therapy); AE (aerobic exercise, such as jogging); RMT (respiratory muscle training); or RT (resistance training).

### Risk of bias assessment

**FIGURE 2:**
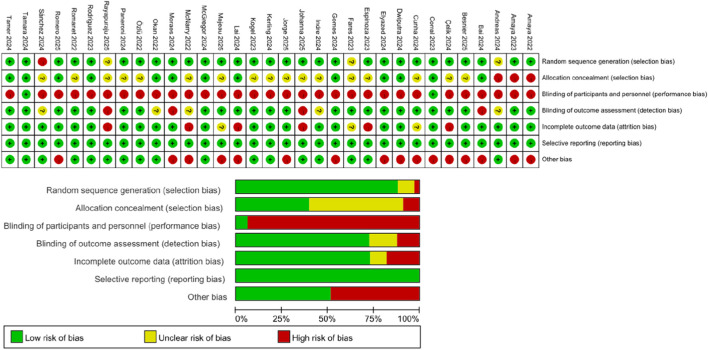
Plot showing the risk of bias.

In the dimensions of random sequence generation (selection bias) and selective reporting (reporting bias), about 80% of the studies were found to be low risk, reflecting standardization of the randomization process and outcome reporting. Most studies did not report allocation concealment measures and were classified as risk uncertain. With regard to participant and personnel blinding, most studies were assessed as high risk because of the inability to blind the intervention (exercise therapy). The blinding methods used for assessment of outcomes were differentiated. Most studies assessed objective indicators (such as pulmonary function tests) in a blinded manner, so they were considered to be low risk for bias. However, some studies used subjective indicators and were deemed to be high risk because they did not clarify the blinding method (red). Incomplete outcomes data are marked in red because of the high rate of loss to follow-up. Some studies were considered high risk because of an imbalance of baseline characteristics or an inadequate sample size (Figure 2).

### Meta-analysis

#### Pulmonary function

**FIGURE 3:**
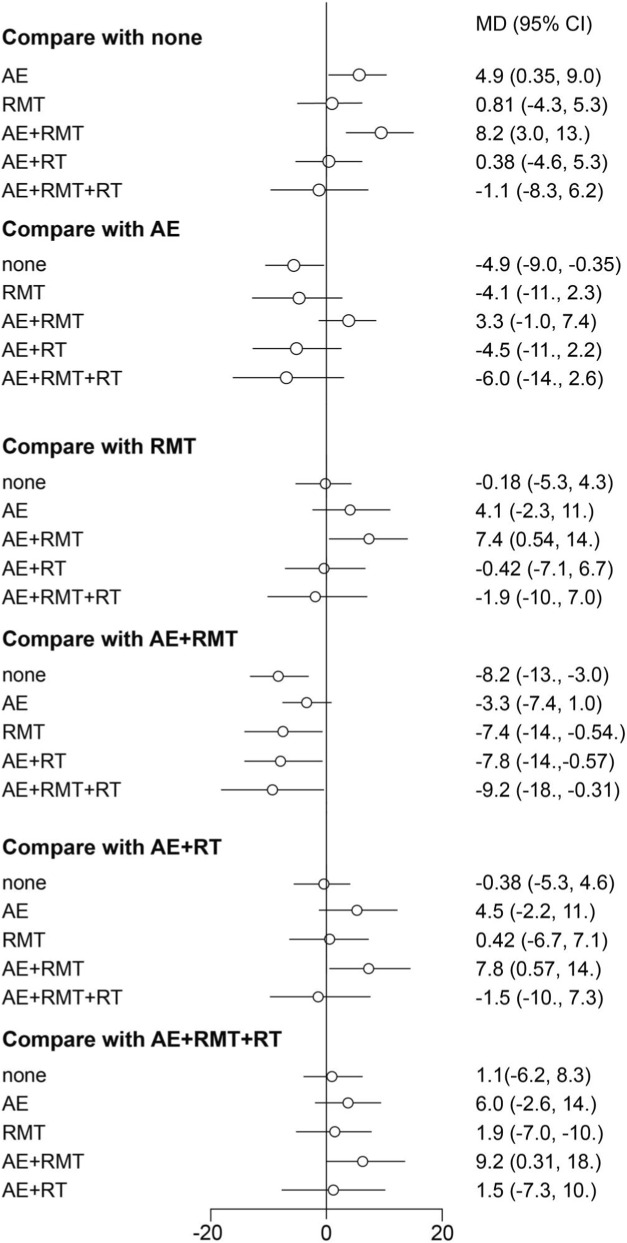
Forest plot showing the results of network meta-analysis of pulmonary function. FEV_1_, forced expiratory volume in 1 s; FVC, forced vital capacity.

Using a Bayesian network, we compared the effects of AE, RMT, AE + RMT, AE + RT, AE + RMT + RT, and no exercise therapy intervention on lung function (forced expiratory volume in 1 s [FEV_1_]/forced vital capacity [FVC]) in patients with PCS. The leverage plot (Supplementary Figure S1) shows that the studies were distributed within the curve, indicating good convergence of the Bayesian model. The surface under the cumulative ranking curve values indicated that the intervention was effective, supported treatment with higher scores, and suggested better FEV_1_/FVC outcomes (Supplementary Figure S2). The top three treatments were AE + RMT (97.62%), AE (76.33%), and RMT (40.62%). The results for the league table (Supplementary Table S2) and the forest plot (Figure 3) indicated that AE + RMT was more effective than the other treatments except for AE, while AE was more effective than no exercise therapy. The network geometry included five nodes representing interventions (None, AE, RMT, AE + RMT, AE + RT, AE + RMT + RT). Edges connecting nodes indicate direct comparisons available in the included RCTs (Supplementary Figure S1). The network was well connected with good model convergence.

#### Peak VO_2_

Peak VO_2_ represents the highest oxygen uptake over a 30-s interval attained during a particular test. Seven of the included studies used peak VO_2_ as an outcome measure. After exclusion of the 2024 study by Kerling et al. (2024), which showed large differences in peak VO_2_ at baseline, we compared the effects of exercise versus non-exercise interventions on the severity of symptoms of PCS in the remaining six randomized controlled trials. The forest plot showed that the experimental group had a statistically significant reduction in peak VO_2_ when compared with the control group (Z = 3.02, P = 0.003) and I^2^ = 0%, suggesting that exercise intervention could effectively improve lung function in patients with PCS (Supplementary Figure S3).

#### Six-minute walk test

Ten studies were included. As shown in Supplementary Figure S8, we divided these studies into two subgroups for analysis. The first subgroup compared the difference in efficacy between exercise intervention and non-exercise intervention. Four studies that compared the difference between AE + RMT + RT and non-exercise intervention were extracted as the second subgroup. The forest plot showed that the experimental group had a statistically significant advantage in terms of improvement in the 6MWT distance when compared with the control group (Z = 6.13, P < 0.00001) (Supplementary Figure S4). Considering the high heterogeneity (I^2^ = 83%), we performed a sensitivity analysis (Supplementary Figure S5), the result for which was relatively robust. For the second subgroup, we found that AE + RMT + RT was more effective than no exercise intervention in the control group (Z = 14.26, P < 0.00001) with I^2^ = 0%, suggesting that the combination intervention of AE + RMT + RT could effectively improve mobility in patients with PCS.

#### Modified medical research council score

The mMRC is used to assess the severity of dyspnea. Six studies were included (Supplementary Figure S10). The forest plot showed that the mMRC score was significant better in the experimental group than in the control group (Z = 2.88, P = 0.004) with I^2^ = 95% (Supplementary Figure S6). Considering the high heterogeneity, we conducted a sensitivity analysis, the results of which suggested that the outcome was relatively robust (Supplementary Figure S7), indicating that exercise therapy can effectively improve dyspnea in patients with PCS.

#### Mental health

Mental health was assessed using the Mental Health (MH) and Mental Component Summary (MCS) tools, both of which are derived from the SF-36 and SF-12. Five studies used MH and five used the MCS as outcome indicators (Supplementary Figures S8, S9). Psychological status was found to be significantly better in the experimental group than in the control group (MH, Z = 2.63, P = 0.008, I^2^ = 0%; MCS, Z = 4.09, P < 0.0001, I^2^ = 10%), suggesting that exercise intervention can effectively improve the mental health status of patients with PCS.

## Discussion

The most common symptom of PCS is fatigue, accompanied by dyspnea and chest pain. Other symptoms include cognitive impairment, palpitations, autonomic dysfunction, joint pain, and muscle pain (Tsuji et al., 2022; Cao et al., 2022; Shafqat et al., 2024; van Doremalen et al., 2020). Mental health is affected when the body is under stress, with many reports of patients under physical stress developing symptoms of anxiety and depression. Moreover, SARS-CoV-2 has a greater ability for immune escape, which may lead to more lasting impairment of the immune system.

The fatigue associated with PCS may be related to blocking of signal transduction as a result of chronic inflammatory invasion. However, a cross-sectional analysis found no significant association between proinflammatory markers and chronic fatigue in most cases. It is possible that a combination of central, peripheral, and psychological factors are involved in the development of post-COVID-19 fatigue. Damage to olfactory neurons leads to increased resistance to drainage of cerebrospinal fluid through the lamina cribrosa, which may contribute to fatigue in patients with PCS. Prolonged exposure to inflammation and cell-mediated immunity leads to decreased metabolism in the frontal lobe and cerebellum, which may also lead to fatigue. Recent studies have shown that long-term social isolation can exacerbate anxiety and depression and promote aging, as can negative psychological and social factors. Finally, peripheral factors, such as direct infection leading to muscle damage, weakness, and muscle fiber and neural-related inflammation, may exacerbate fatigue (Cao et al., 2022; Shafqat et al., 2024; Mehandru and Merad, 2022; Scharf and Anaya, 2023).

Dyspnea is also a common symptom of PCS. Pulmonary dysfunction, indicated by abnormalities in pulmonary function, can still be observed at 1 month after discharge in patients hospitalized with COVID-19 (Carfi et al., 2020; Mandal et al., 2021; Halpin et al., 2021). A cohort study found that many patients with COVID-19 had cardiac involvement, persistent myocardial inflammation, and elevated serum troponin levels 71 days after diagnosis (Carfi et al., 2020; Puntmann et al., 2020). The main reason for the cardiac involvement is that the ACE2 receptor is strongly expressed in the human heart, providing a direct route for SARS-CoV-2 infection (Perez-Bermejo et al., 2020).

A similar study found that cognitive scores at 3 months after discharge were significantly lower, similar in 40% of patients to those with moderate traumatic brain injury, and similar in 26% of cases to those in patients with mild Alzheimer’s disease. This finding may be related to long-term social isolation. The COVID-19 epidemic has had a negative impact on people’s mental health. During recovery from the acute infection, people may develop longer-term psychiatric symptoms, including post-traumatic stress disorder, depression, anxiety, and obsessive-compulsive symptoms, which seriously impede recovery from PCS. People with dementia become more depressed, anxious, agitated, and lonely (Velayudhan et al., 2020). This may be related to invasion of the virus into neurons within the central nervous system, producing neuroinflammation and chronic degenerative changes. SARS-CoV-2 may also affect the permeability of the blood-brain barrier, allowing peripheral cytokines and other blood-derived foreign bodies to enter the central nervous system and exacerbate neuroinflammation (Romero-Sanchez et al., 2020).

A scientifically designed exercise program can be used to prevent, treat, or promote rehabilitation in patients with a variety of diseases. Exercise is widely used in the management of chronic disease and rehabilitation of sports injuries and to improve mental health. Exercise therapy includes AE, respiratory exercise, and resistance exercise and has been confirmed to aid recovery from illness. Initially, it was used mainly in patients recovering from conditions affecting the bones and joints, limbs, spine, and nervous system. In-depth studies have found that exercise therapy is an effective intervention for many diseases affecting the internal organs, and there is ample evidence of improvement in patients with psychiatric conditions. In terms of immune regulation, moderate exercise can upregulate the activity of natural killer cells, neutrophils, and macrophages and improve the detection and clearance of viruses. Studies have found that regular exercise reduces the risk of respiratory infections by 40%–50% (Nieman et al., 2011). AE can promote the proliferation of T cells (especially CD4^+^ Th1 cells) and memory B cells, which may accelerate the specific immune response to SARS-CoV-2. Long-term resistance exercise can reduce levels of inflammatory markers, such as IL-6 and tumor necrosis factor-alpha, which is an important way to alleviate the “cytokine storm” caused by COVID-19 (Kishimoto and Kang, 2022). Various exercises can improve antioxidant capacity, reduce levels of reactive oxygen species, and increase expression of malondialdehyde to varying degrees. It can also promote mitochondrial biosynthesis and improve energy metabolism disorders caused by COVID-19 (Varda et al., 2022). Considering that ACE2 is a viral receptor, some investigators have hypothesized that exercise may reduce viral invasion by regulating the expression and distribution of ACE2. Animal studies have also shown that exercise upregulates the circulating soluble form of ACE2 or competitively inhibits viral binding (Driggin et al., 2020). Recent aging-related phenotypes have incorporated psychosocial isolation, with patients who have PCS having more or less reduced social contact, which greatly slows disease recovery and increases mental distress. Respiratory exercise can reduce cortisol, increase β-endorphins, regulate immunity through the hypothalamic–pituitary–adrenal axis, and alleviate the suppression of immunity by COVID-19-related emotional stress (Pizzorno, 2023). AE can also promote the release of endorphins and serotonin, improve the patient’s mental health, restore a sense of control over the body, and reduce “fear of disease” (de Oliveira et al., 2022).

## Limitations

The results of this meta-analysis of the existing literature underscore the effectiveness of exercise therapy in patients recovering from COVID-19. However, which type of exercise is most effective and its exact content have not been clarified, and whether there is a difference in effect between long-term exercise and short-term exercise has not been explored. Another problem worth considering is the lack of consistency in the evaluation criteria used. These criteria vary according to the type of exercise, which is an impediment to further research in this area. Potential publication bias cannot be completely excluded, as most included studies were small-scale and published in peer-reviewed journals. We found that most of the relevant studies in SportDiscus database were already included in the four databases we explored. However, this database better reflects the professionalism of exercise therapy, so we will take full account of its content in future research to explore the advantages of exercise therapy. In addition, long-term follow-up data were lacking, and there was variability in the definitions of PCS and outcome measures across studies.

## Prospects

In the future, we will explore the effect of exercise therapy on the course of PCS further in the hope of formulating consistent evaluation criteria and developing an efficient method for assessment of cognitive function, emotional state, physical function, cardiopulmonary status, and quality of life in these patients. The ultimate goal will be to identify the best treatment strategy and explore the effects of combined exercise based on multimodal intervention.

## Conclusion

The severity of the symptoms of COVID-19 are lessening with the passage of time but still cannot be ignored. The existing treatments are symptomatic only, and there is no cure for this illness. Therefore, patients need to engage with other rehabilitation strategies. Exercise is becoming increasingly popular as a convenient and effective treatment. Using a seven-item data scale, we have confirmed that exercise therapy can significantly improve lung function, physical activity status, and emotional status in patients with PCS. Combined with the existing evidence, we believe that exercise is a safe and effective treatment for these patients.

## Data Availability

The original contributions presented in the study are included in the article/Supplementary Material, further inquiries can be directed to the corresponding authors.
