# Supplementary material for: Clinical efficacy of exercise in the treatment of post-COVID-19 syndrome: a systematic review and network meta-analysis
Source: Front Physiol. 2025 Dec 18;16:1656713. doi: 10.3389/fphys.2025.1656713 (PMC12756122; doi:10.3389/fphys.2025.1656713)
Supplement: Supplementary file 1 [file DataSheet1.docx]

**Supplementary Materials**


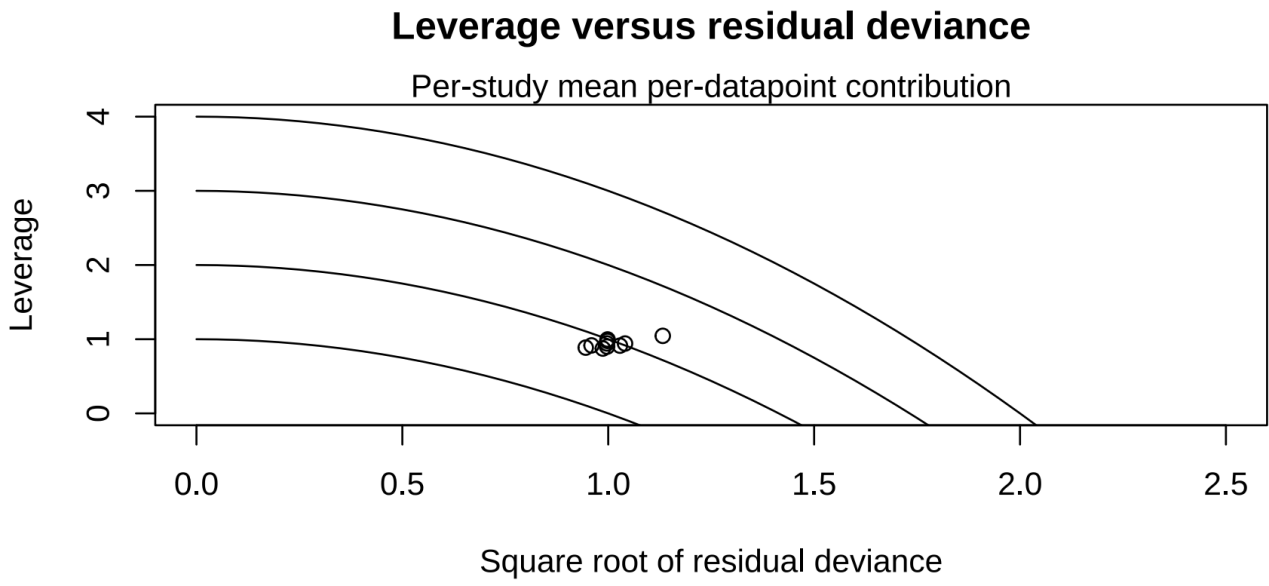


Fig. S1. Leverage plot showing the distribution of studies within the curve.


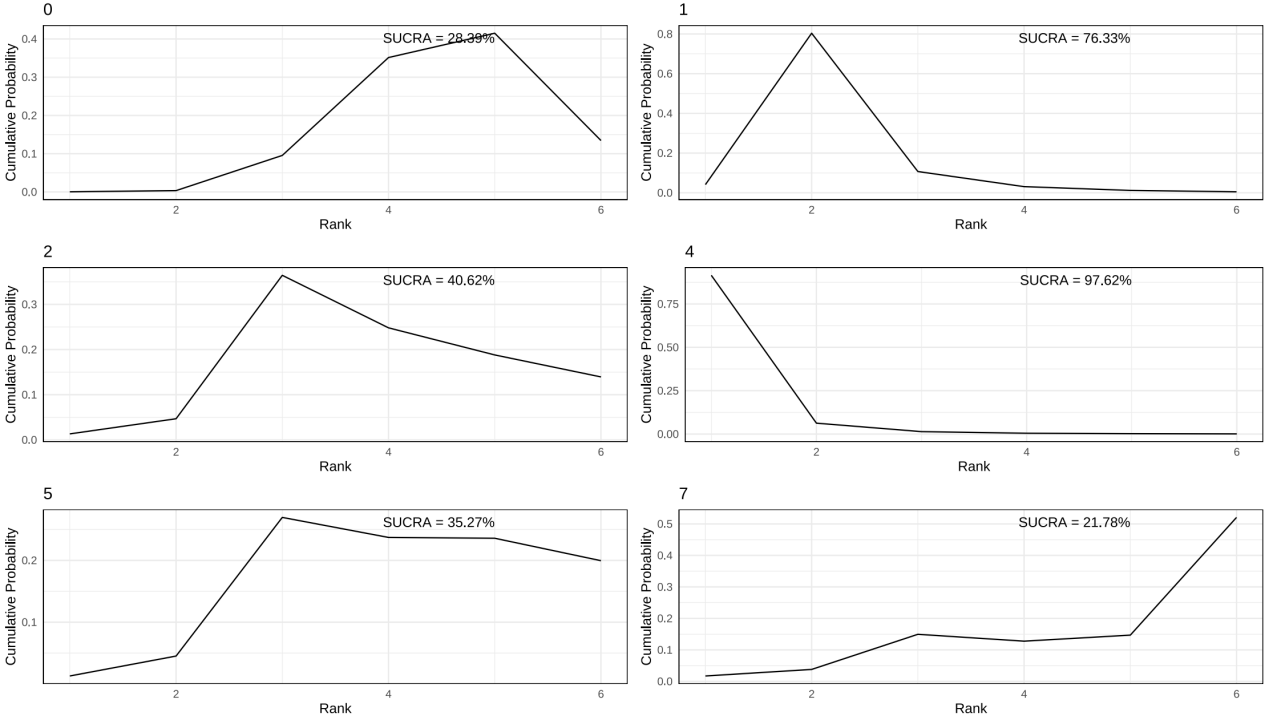


Fig. S2. Probability ranking diagram. 0, None; 1, AE; 2, RMT; 4, AE+RMT; 5, AE+RT; 7, AE+RMT+RT. AE, aerobic exercise; None, no exercise therapy; RT, resistance muscle training; RMT, respiratory muscle training


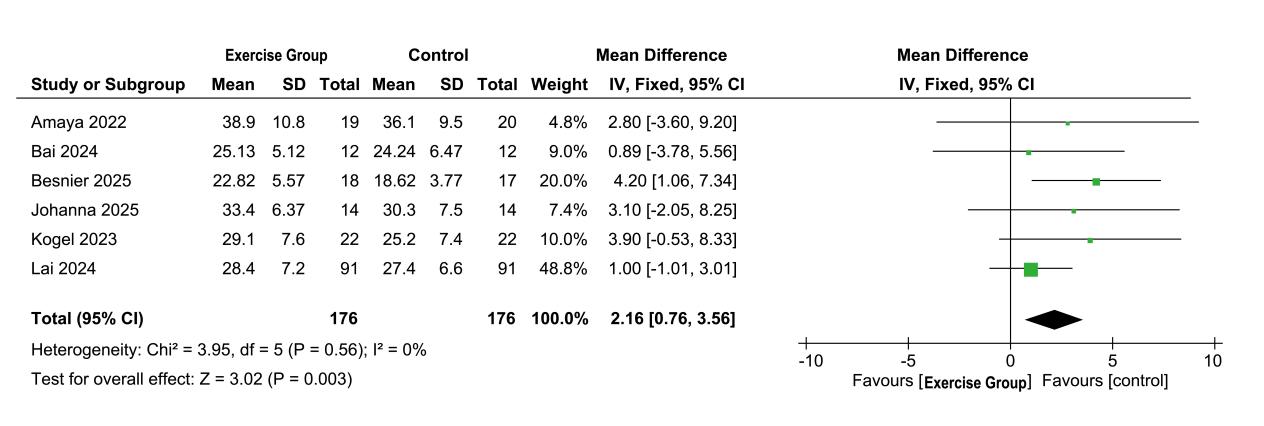


Fig. S3. Results of meta-analysis for peak VO_2_.


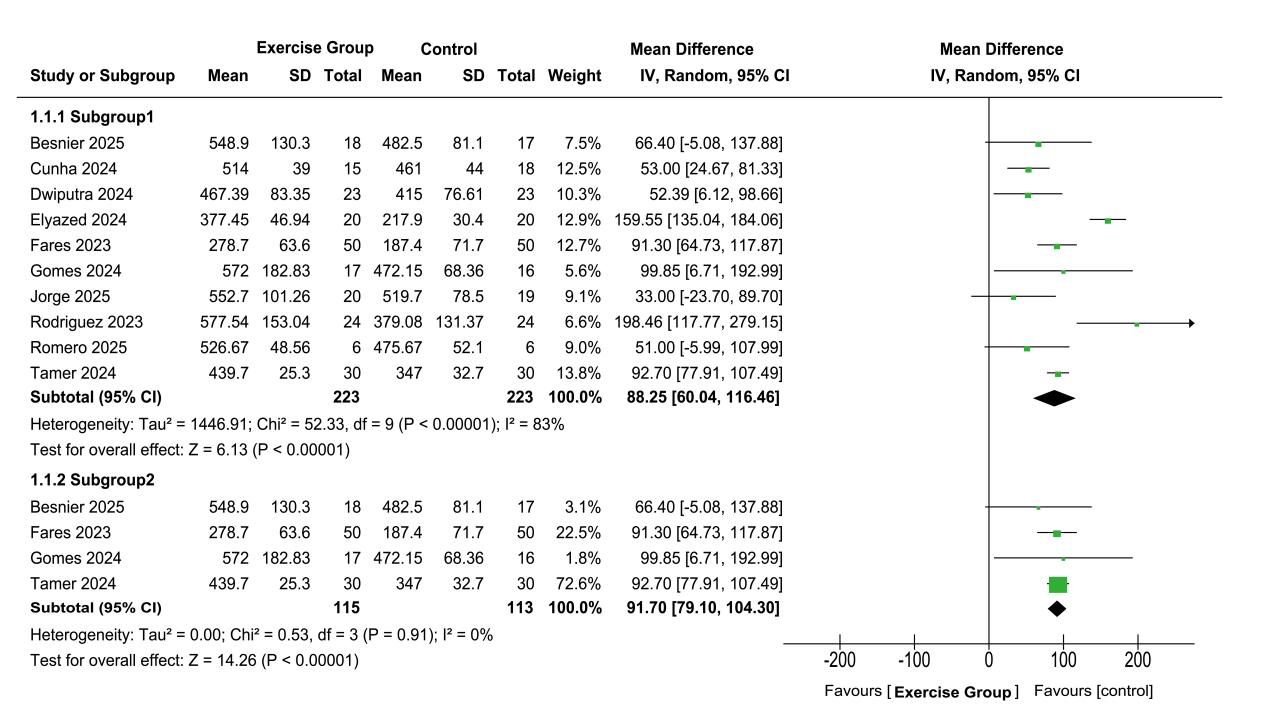


Fig. S4. Results of meta-analysis for the six-minute walk test.


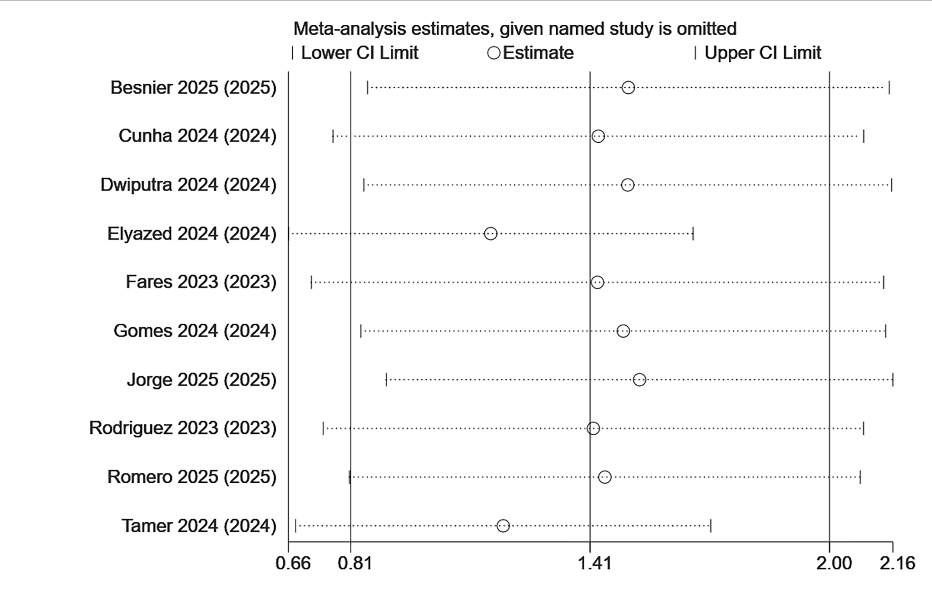


Fig. S5. Results of sensitivity analysis for the six-minute walk test.


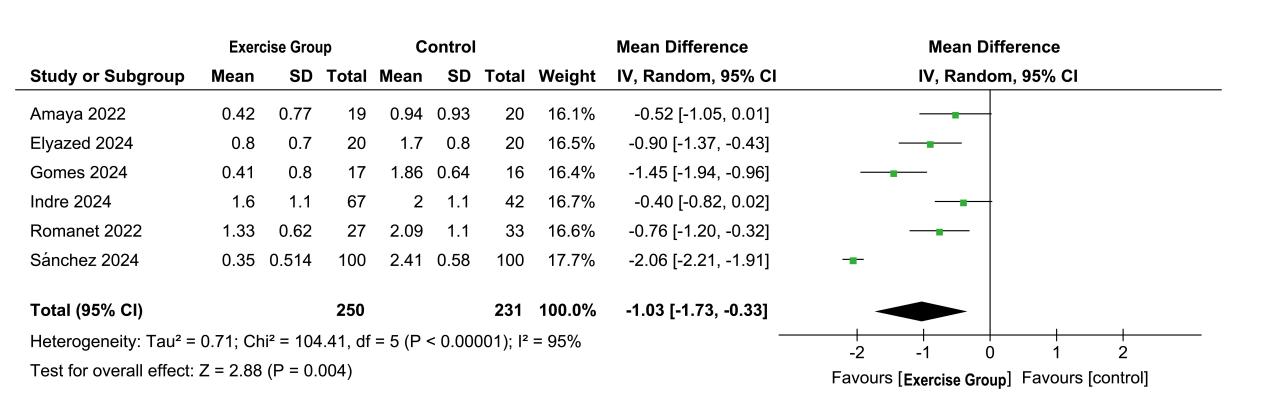


Fig. S6. Results of meta-analyses for the modified Medical Research Council score.


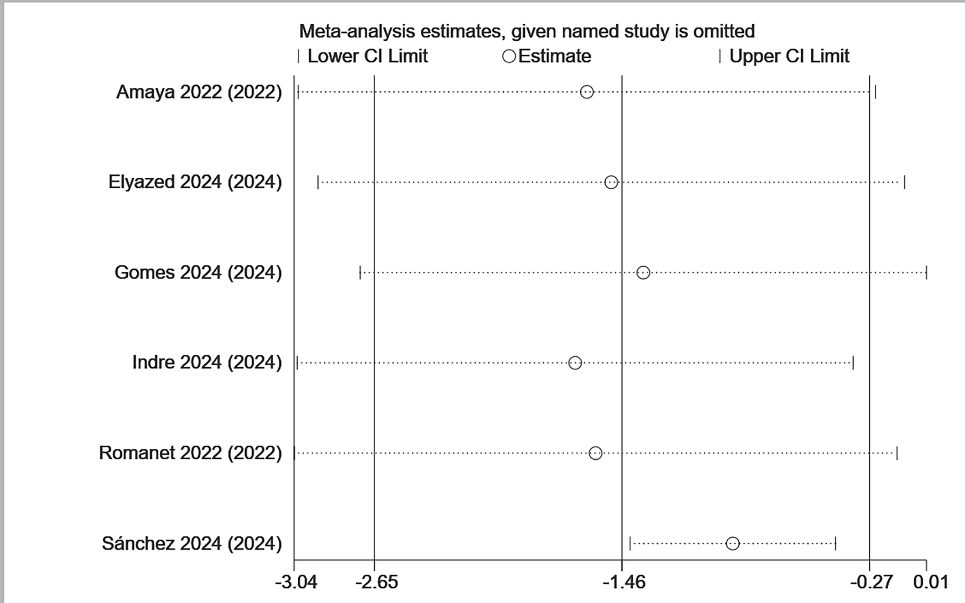


Fig S7 Results of sensitivity analysis for the modified Medical Research Council score.


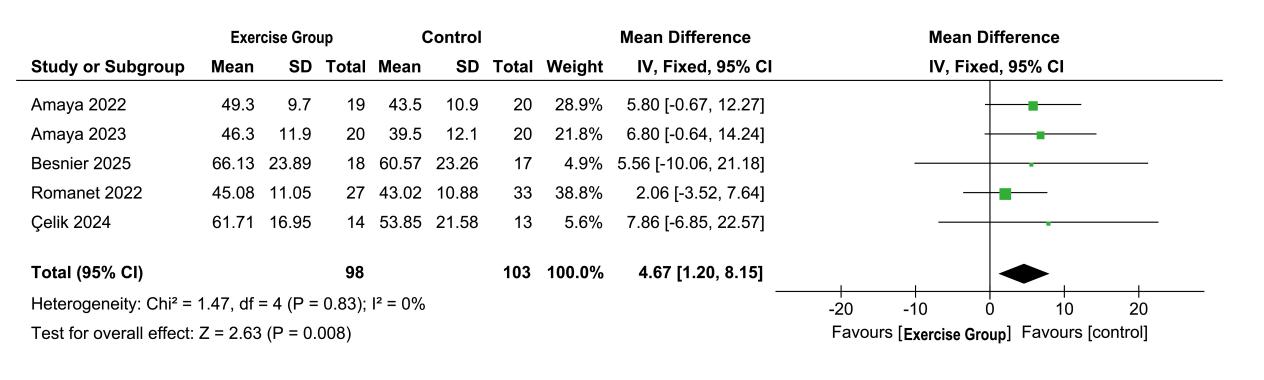


Fig S8 Results of meta-analyses for the Mental Health score.


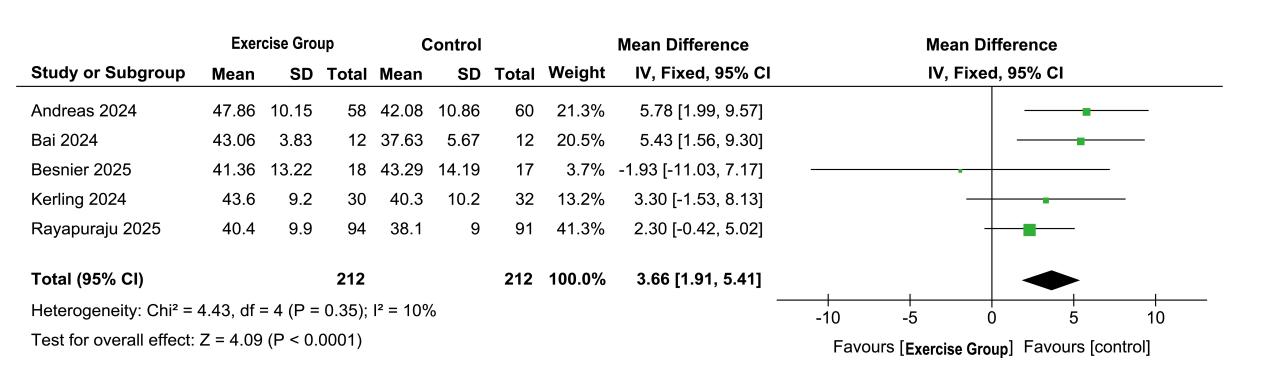


Fig S9 Results of meta-analyses for the Mental Component Summary score.

Table S1 Characteristics of studies included in the meta-analysis

1MSTS, 1-minute-sit-to-stand; 6MWT: 6-minute walk test; 30STST, 30-second sit-to-stand test; AE, aerobic exercise; BDI, Baseline Dyspnea Index; BORG-M, modified Borg Perceived Exertion Scale; CCQ, Clinical COPD Questionnaire; CEQ, Credibility Expectancy Questionnaire; CFQ11, Chalder Fatigue Scale; CPET, cardiopulmonary exercise test; DS, depressive symptoms; EQ-5D-5L, Euroqol-5 dimensions-5 levels; FAS, Fatigue Assessment Scale; FIT, Fatigue Index Time; FSS, Fatigue Severity Scale; GDS, Geriatric Depression Scale; HADS, Hospital Anxiety and Depression Scale; HP, health perception; HRQoL, health-related quality of life; HRV, 5-min short term heart rate variability; ICF, International Classification of Functioning; IES-R, 16 Severity of Post-Traumatic Stress Disorder; IPAQ, International Physical Activity Questionnaire Short-form; ISI, Insomnia Severity Index; K-BILD, 15-item King’s Brief Interstitial Lung Disease questionnaire; mBS, modified Borg Scale; MD12, Multidimensional Dyspnea-12; MCID, minimal clinically important difference; MDP, Multidimensional Dyspnea Profile; MIP, maximal inspiratory pressure; mMRC, modified Medical Research Council; None, control group without exercise therapy; PCFS, post-COVID-19 Functional State Scale; peak VO_2_, highest oxygen uptake over a 30s interval attained during a particular test; PF, pulmonary function; PFI, Physical Fitness Index; PHQ-9, Patient Health Questionnaire-9; POMS, Profile of Mood States; PSQI, Pittsburgh Sleep Quality Index; PSS, Perceived Stress Scale; QoL, quality of life; RMT, respiratory muscle training; RPE, Rate of Perceived Exertion; RT, resistance training; SF-36, Short-Form 36 Health Survey Questionnaire; SGRQ, St George’s Respiratory Questionnaire; SF-12, 12-Item Short-Form Survey; SMIP, sustained maximal inspiratory pressure; SQ, sleep quality; SS8, Somatic Symptoms Scale-8; SSTQ, the Singapore Smell and Taste Questionnaire; STAI, State-Trait Anxiety Inventory; TDI, Transition Dyspnea Index; TUG, timed up-and-go; VAFS, Visual Analog Fatigue Scale; VAS, visual analog scale; WHO-QOL-BREF, WHO-quality of life-BREF


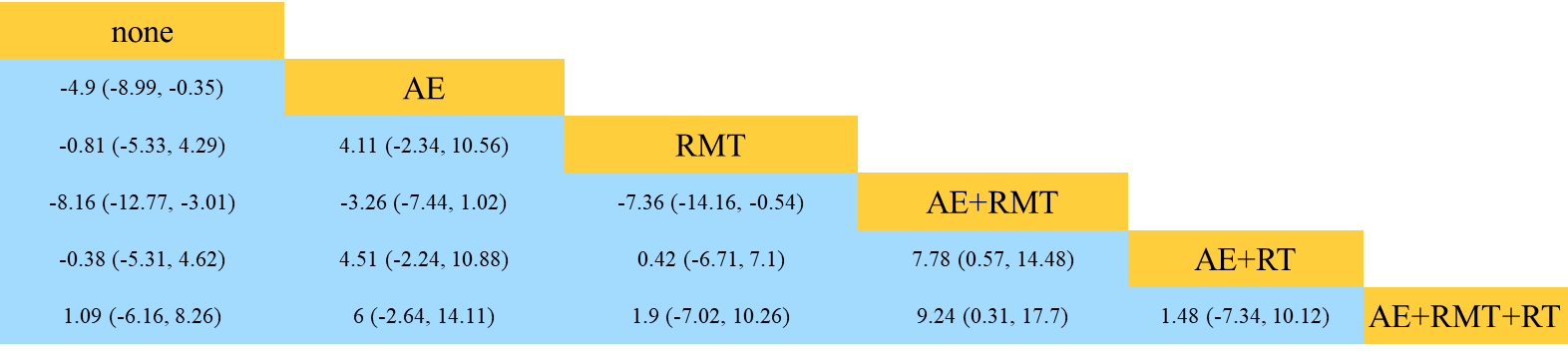


Table S2 League table
